# Sampling of urine for diagnosing urinary tract infection in general practice – First-void or mid-stream urine?

**DOI:** 10.1080/02813432.2019.1568708

**Published:** 2019-01-28

**Authors:** Hoelmkjaer Pernille, Bjerrum Lars, Mäkelä Marjukka, Siersma Volkert, Holm Anne

**Affiliations:** a Research Unit for General practice and Department of General Practice, University of Copenhagen, Copenhagen, Denmark;; b Department of Health and Social Care Systems, National Institute for Health and Welfare, Helsinki, Finland

**Keywords:** “Urinary tract infections” [Mesh] “Urine” [Mesh], “Specimen handling” [Mesh] “Urine specimen collection” [Mesh] “Primary health care” [Mesh] “General practice” [Mesh]

## Abstract

**Objective:** To study 1) whether the accuracy of point-of-care (POC) urine tests (dipstick, phase-contrast microscopy and culture) differs when performed on first-void urine (FVU) compared to mid-stream urine (MSU), and 2) if a delay of analysis up to four hours affects the accuracy of POC tests when performed on urine from symptomatic of urinary tract infection (UTI), non-pregnant women in general practice.

**Design:** Prospective diagnostic study using paired samples.

**Setting/Intervention:** Three general practices in Copenhagen. Each woman delivered FVU and MSU samples from the same void. As a reference standard, 8 ml of MSU was sent for culture at the Microbiology Department.

**Patients:** 117 women with one or more symptoms of UTI.

**Main outcome measures:** Sensitivity, specificity and agreement with the reference standard of FVU and MSU with different time delays (zero vs. one vs. four hours) as compared to reference standard (MSU at time zero in boric acid tubes).

**Results:** All three POC tests performed on MSU were significantly more in agreement with the reference than when performed on FVU when analysis was done immediately. The error rate was 16% for MSU vs. 23% for FVU with POC culture, 27% vs. 40% with microscopy and 25% vs. 33% with dipstick testing. Delay of analysis up to four hours did not decrease agreement with the reference.

**Conclusion/Implication:** MSU samples should be used in general practice for optimal accuracy of POC tests. Analysis can be delayed up to four hours.

**Key points**
- Point-of-care tests (dipstick testing, microscopy and culture) for diagnosing urinary tract infection performed on mid-stream urine samples are significantly more accurate than when performed on first-void urine samples.
- Delay of analysis up to four hours did not decrease the accuracy of any of the point-of-care tests.
- Point-of-care culture was more accurate than dipstick and microscopy both when performed on mid-stream urine and first-void urine
- The main contaminant in first-void urine samples was Enterococci spp., which contributed to the majority of false positives.

## Introduction

Urinary tract infection (UTI) is a common reason for encounter in general practice and urine samples are handled daily by both general practitioners (GPs) and practice personnel [1]. Typical uropathogens, e.g. *E. Coli* and Enterococci spp. may act as contaminants [2]. Features like high BMI, days since last shower or the degree of intimal hair removal could theoretically contribute to this contamination. Contaminated urine samples may result in diagnostic misclassification, overtreatment, unnecessary side-effects and antibiotic resistance [3,4].

In Denmark, most patients suspected for UTI are instructed to deliver a mid-stream urine (MSU) sample by voiding firstly into the toilet and secondly into the urine container. Practice personnel are trained to analyze the sample shortly after urination by point-of-care (POC) testing, which may include urine dipstick, microscopy and culture. The recommendation to use MSU is based on research done at hospitals with a much higher prevalence of UTI than in primary care. Instructing patients in MSU can be time-consuming and challenging, leaving interpretation of POC tests difficult. A systematic review suggested that sampling technique may not affect accuracy of urine culture performed on urine from symptomatic patients in primary care, but none of the included studies compared the accuracy based on paired urine samples techniques [5]. If the urine sample is left at room temperature beyond four hours before analysis it may lead to increased number of colony forming units (CFU) interpreted as significant growth. However, no studies have investigated delays of up to four hours before analysis. Furthermore, studies of the consequences of delay are quite old and conducted before the thresholds for significant growth were lowered to the current cut-offs [6–8]. Because the threshold for significant growth has been lowered, delayed urine analysis could affect the accuracy of POC testing as well.

In Danish general practice, antibiotic treatment is usually initiated based on clinical history combined with POC diagnostics. Waiting on results from the hospital may cause unnecessary delay of up to four days, which is unacceptable for most patients [9].

The aims of the study were to investigate the influence of 1) sampling technique (First-void urine(FVU) vs. MSU) and 2) delay of analysis (zero vs. one vs. four hours) on the accuracy of POC tests (culture, phase-contrast microscopy and urine dipstick) for UTI in symptomatic non-pregnant women with suspected UTI in general practice. The reference standard for both aims was urine culture performed on MSU at the microbiological department where bacterial multiplication was stabilized by adding boric acid immediately after sampling.

## Material and methods

### Study design

Prospective diagnostic study using paired samples.

### Setting and recruitment of patients

Patients presenting with symptoms of UTI in three general practices in Copenhagen were included. Women were eligible if they were 18 or older, non-pregnant and presented one or more symptoms of UTI (dysuria, frequency or urge). Exclusion criteria were recent bladder surgery, urinary tract abnormality or not being able to deliver enough urine to make two urine samples from the same portion of urine. The women were orally informed about the study, presented with written material and asked to sign a consent form. The involved clinics were located in Copenhagen and covered a total of approximately 18,000 listed patients.

### Data collection

Upon inclusion, clinical information was collected using a structured case-report form. See Table 1 for list of covariates.

**Table 1.** Baseline data (*N* = 117).

	*n* / (%)
Age	
30 or below	58 (50)
31 to 60	46 (39)
61 or older	13 (11)
History of vaginal deliveries	
Nulliparous	75 (64)
Body-mass index (self-reported)	
Below 25	93 (79)
25–30	16 (14)
30 or above	8 (7)
Duration of symptoms	
Less than 4 days	55 (47)
Between 4 and 6 days	15 (13)
7 days or more	47 (40)
Urine incubation time in bladder	
1 hour or less	69 (59)
1–4 hours	41 (35)
4 hours or more	7 (6)
Days since latest shower	
0	65 (56)
1 or more	52 (44)
Genital hair removal	
All removed	45 (38)
Some removed	18 (15)
Nothing removed	54 (46)
Reference culture result	
Significant growth (positive sample)	52 (44)
*E. coli* – out of total *	41 (35)

* Of the 52 positive samples, 79% were due to E.coli.

### Urine samples

The included women were instructed to deliver a first-void urine (FVU) sample in one cup, squeeze off, and a mid-stream urine (MSU) sample in a second cup. Hence, each woman delivered two urine samples. Both samples were placed at room temperature and analyzed immediately after urination (time zero), after one hour and after four hours. First author (PB) processed all samples.

### Reference standard

A few milliliters of all MSU-samples were transferred to a boric acid tube and sent to Hvidovre Hospital Microbiology Department for culture. Urine samples were analyzed on Inoqul ATM Bi-plate (CHROMagar and blood agar) with 10 µL on each half of the agar [10,11]. All samples were quantified. Significant growth was defined as growth of ≥10^3^ CFU/mL for *E. coli* and *S. saprophyticus*, ≥10^4^ CFU/mL for other typical uropathogens and ≥10^5^ CFU/mL for possible uropathogens in accordance with European consensus [8]. Growth of more than two different colonies (mixed cultures) was considered as non-significant growth.

### POC tests

#### POC culture

10 µl of urine from each urine sample (FVU and MSU) was inoculated on two separate agars (Flexicult ID® SSI Diagnostica) and the number of colonies was counted the following day. The Flexicult ID® is a chromogenic agar allowing identification and quantification of most primary and secondary uropathogens. The culture was interpreted according to European guidelines [8]. More than five colonies corresponded to 10^3^ CFU/mL and was interpreted as a positive result for a primary uropathogen. For secondary uropathogens, more than 50 colonies corresponded to 10^4^ CFU/ml was interpreted as a positive result.

#### Microscopy

A drop of uncentrifuged urine from each sample was analyzed by an Olympus phase-contrast microscopy at 400 times magnification. Urine samples were classified as positive if there were one or more bacteria **or** four or more leucocytes per field of vision **unless** there were ≥3 squamous cells or ≥3 different microorganisms present. In this case the sample was labeled as contaminated.

#### Urine dipstick

The first (*n* = 25) were analyzed by Combur 5 ® and interpreted visually. The rest (*n* = 92) were analyzed by a semi-automated urine analyzer Urisys 1100 ®. Dipstick analyses were considered positive if there was a positive reaction for nitrite (≥+) or a positive reaction (≥++) for leucocytes [12].

### Blinding and timing

PB was blinded to the result of the dipstick test when performing microscopy and to the result of the culture performed at the microbiological department when interpreting the results of urine dipstick, phase-contrast microscopy and culture performed in general practice. All POC cultures were photographed and sent for a second evaluation by the last author (AH) who was blinded to clinic information about patients and results of urine analyses. Discrepancies between the two evaluations done by PB and AH were discussed, and the colonies were recounted in case of doubt.

### Statistical analysis

We assessed agreement of the POC tests performed on MSU at time zero to be 90% according to previous studies [13]. In a power calculation we determined that we needed samples from 125 patients to detect a drop in accuracy from 90% to 80% due to sampling technique or delay of analysis with 80% probability, assuming an intra-class correlation of 0.2 between the samples from the same patient; this with a significance level of 5%

Agreement between the result of POC tests performed on urine from the two different sampling techniques and the three different time-delays with the external reference culture was done using a paired logistic regression model taking into account paired samples. A Wald test was performed to evaluate the difference in agreement. These analyses were done separately for each sampling modality and at each of the time delays.

To see whether third variables (listed in Table 1) influenced the effect of sampling technique on agreement with the reference, the interaction between these third variables and the variable indicating the sampling technique was added to the above logistic regression models and tested with a Wald test. This model was also used to see if manual reading versus automated reading of the dipsticks influenced the effect of sampling technique on agreement with the reference. The accuracy (sensitivity and specificity) was calculated for both sampling techniques and all time-delays. A p-value of 0.05 was considered significant. The statistical analysis was done in SAS v.9.4

## Results

The study was conducted from September 2015 to June 2016 and a total 122 women were eligible for participation. Two were excluded due to not being able to deliver a sufficient amount of urine and three were excluded due to already having participated in the study leaving 117 women as eligible for inclusion. PB handled all samples and there was no missing data.

Table 1 shows the baseline data. Participants were generally slim, with a low BMI, good hygiene and evenly divided whether they had any hair removal or symptoms for less than six days. Only a small percentage managed to have urine in the bladder for four hours prior to urinating.

The columns show overall agreement with the reference for each POC modality and sampling technique at the three different time-points for delay of analysis. The right column shows the significance-level of the effect of sampling technique on overall agreement with the reference. The row below each point-of-care modality shows the significance-level of the effect of delay of analysis on overall agreement with the reference. The reference for all analyses is culture performed in the microbiological department on MSU incubated in boric acid immediately after voiding. *N* = 117. *p-value obtained from logistic regression model.

Table 2 shows the agreement of the three POC tests with the reference standard at the three time-points.

**Table 2.** Overall agreement of urine POC test modalities as compared to reference (*N* = 117).

	Agreement with reference standard n (%)						
	Both FVU and MSU	Only MSU	Only FVU	Neither FVU nor MSU	Overall agreement, MSU	Overall agreement, FVU	*p*-value (sampling method)*
POC culture							
Immediate analysis	89 (76)	9 (8)	1 (1)	18 (15)	98 (84)	90 (77)	.03
Analysis after 1 hour	87 (74)	11 (9)	1 (1)	19 (16)	98 (84)	88 (75)	.003
Analysis after 4 hours	91 (78)	7 (6)	4 (3)	15 (13)	98 (84)	95 (81)	NS
p-value (time delay)*					NS	NS	
Urine dipstick							
Immediate analysis	73 (63)	14 (12)	5 (4)	25 (21)	87 (74)	78 (67)	.04
Analysis after 1 hour	74 (63)	13 (11)	6 (5)	25 (21)	87 (74)	80 (68)	NS
Analysis after 4 hours	74 (63)	11 (9)	9 (8)	23 (20)	85 (73)	83 (71)	NS
p-value (time delay)*					NS	NS	
POC microscopy							
Immediate analysis	63 (54)	22 (19)	7 (6)	25 (21)	85 (73)	70 (60)	.005
Analysis after 1 hour	70 (60)	13 (11)	12 (10)	22 (19)	83 (71)	82 (70)	NS
Analysis after 4 hours	83 (71)	13 (11)	4 (3)	18 (15)	96 (82)	87 (74)	.03
p-value (time delay)*					0.0045	0.0004	

MSU: mid-stream urine; FVU: first-void urine; POC: Point of Care; NS: not significant.

For POC culture, about 76% of tests were in agreement with the reference both when the test was performed on MSU and FVU irrespective of time delay. About 15% of the tests were not in agreement with the reference neither when performed on MSU nor on FVU irrespective of time delay. In 8% of the samples, only MSU agreed with the reference compared to 2% were only FVU agreed. This resulted in a significantly higher overall agreement with the reference of POC culture performed on MSU than POC culture performed on FVU at immediate analysis (*P* = .03) and at one hour time delay (*P* = .003). The difference was not significant at four hours delay. There was no significant effect of delay of analysis on agreement with the reference of POC culture neither when performed on MSU nor on FVU.

For urine dipstick analysis 63% of the tests were in agreement with the reference both when performed on MSU and FVU and 21% of test were not in agreement with the reference neither when performed on MSU nor on FVU. 11% of tests were only in agreement with the reference when performed on MSU compared to 6% when performed on FVU. This resulted in a significantly higher agreement with the reference of dipstick analysis performed on MSU than on FVU at immediate analysis (*P* = .04), but the difference was not significant at one and four hours delay. There was no significant effect of delay of analysis for urine dipstick.

For microscopy there was a significant effect of delay of analysis for both MSU (*P* = .0045) and FVU (*P* = .0004) with both modalities increasing their agreement with the reference with increasing time delay. The overall agreement with the reference was significantly better when microscopy was performed on MSU than FVU at immediate analysis (*P* = .005) and at 4 hours time delay (*P* = .03) but the difference was not significant at one hour time delay.

The columns show sensitivity and specificity for each POC modality and sampling technique at the three different time-points for delay of analysis. The reference for all analyses is culture performed in the microbiological department on MSU incubated in boric acid immediately after voiding. *N* = 117

Table 3 shows sensitivity and specificity of the three POC tests when performed on MSU and FVU at all time delays. Sensitivity of POC culture based on both FVU and MSU was nearly 90% irrespective of delay. Specificity of POC culture based on MSU was about 80% regardless of time delay, while specificity of POC culture based on FVU was lower, ranging from 63 to 75%.

**Table 3.** Sensitivity and specificity of urine POC test modalities as compared to reference.

	Sensitivity proportion/(95% CI)		Specificity proportion/(95% CI)		Overall agreement proportion/(95% CI)	
	MSU	FVU	MSU	FVU	MSU	FVU
POC culture						
Immediate analysis	0.88 (0.80–0.97)	0.90 (0.82–0.98)	0.80 (0.70–0.90)	0.68 (0.56–0.79)	0.84 (0.77–0.90)	0.77 (0.70–0.85)
Analysis after 1 hour	0.88 (0.80–0.97)	0.88 (0.80–0.97)	0.78 (0.68–0.88)	0.63 (0.51–0.75)	0.84 (0.76–0.90)	0.75 (0.66–0.82)
Analysis after 4 hours	0.87 (0.77–0.96)	0.89 (0.80– 0.97)	0.82 (0.72–0.91)	0.75 (0.65– 0.86)	0.84 (0.77– 0.90)	0.81 (0.74–0.88)
Urine dipstick						
Immediate analysis	0.73 (0.59–084)	0.81 (0.67–0.90)	0.75 (0.63–0.85)	0.55 (0.43–0.68)	0.74 (0.66–0.82)	0.67 (0.58–0.75)
Analysis after 1 hour	0.69 (0.57–0.82)	0.73 (0.61–0.85)	0.78 (0.68–0.88)	0.65 (0.53–0.76)	0.74 (0.66–0.82)	0.68 (0.60–0.77)
Analysis after 4 hours	0.65 (0.52–0.78)	0.79 (0.68–0.90)	0.78 (0.68–0.88)	0.65 (0.53–0.76)	0.73 (0.65–0.81)	0.71 (0.63–0.79)
POC microscopy						
Immediate analysis	0.61 (0.47–0.75)	0.69 (0.55–0.81)	0.82 (0.70–0.90)	0.52 (0.50–0.65)	0.73 (0.65–0.81)	0.60 (0.51–0.69)
Analysis after 1 hour	0.58 (0.44–0.71)	0.77 (0.65–0.88)	0.81 (0.72–0.91)	0.65 (0.53–0.76)	0.71 (0.63–0.79)	0.70 (0.62–0.78)
Analysis after 4 hours	0.71 (0.59–0.83)	0.75 (0.63– 0.87)	0.91 (0.84–0.98)	0.74 (0.63– 0.85)	0.82 (0.75– 0.89)	0.74 (0.66–0.82)

MSU: mid-stream urine; FVU: first-void urine; POC: Point of Care; NS: not significant.

For POC microscopy, the sensitivity was generally lower (61–71% for microscopy based on MSU and 69–77% for microscopy based on FVU). Specificity for microscopy based on MSU ranged from 81–91% and on FVU from 52 to 74%. Urine dipstick had a sensitivity of 65–73% based on MSU and 73–81% based on FVU and a specificity of 75–78% based on MSU and 55–65% based on FVU.

The result of the urine dipstick analysis of the 92 women whose urine dipstick analysis was performed on the Urilyzer compared to those whose dipstick was read manually. It showed that the accuracy of the test was significantly worsened (odds ratio (OR) 3.30 [95%CI 1.44–7.60]; *p* = .0049, i.e.) by manual reading. Especially specificity was lower when read manually. However, manual reading did not significantly influence the effect of sampling technique on agreement with the reference.

None of the variables shown in Table 1 (age, history of vaginal deliveries, body-mass index, duration of symptoms, urine incubation time in bladder, days since latest shower and degree of genital hair removal) significantly influenced the effect of sampling technique on agreement with the reference.

Nine patients had a false-positive POC culture performed on FVU, but true-negative POC culture performed on MSU at time zero. The bacteriological characteristics of these patients are listed in Table 4. Three of these patients had a true-neagtive POC culture performed on FVU after four hours time-delay. Enterococcus spp. were responsible for false-positive results at time zero in six cases.

**Table 4.** Characteristic of false positive tests from first-void urine at time zero (*n* = 9).

Patient	Uropathogens identified in POC cultureat immediate analysis	Quantity (cfu/mL)
1	Enterococcus spp.	10^4^
	Skin bacteria	10^3^
2	*E.coli*	<10^3^
	Enterococcus spp.	10^4^
	Skin bacteria	10^3^
3	Enterococcus spp.	10^4^
	Skin bacteria	10^3^
4	*E.coli*	10^4^
	Enterococcus spp.	10^5^
	Skin bacteria	10^3^
5	*E. coli*	10^3^
	Enterococcus spp.	<10^3^
6	*E. coli*	10^3^
	Enterococcus spp.	10^3^
	Skin bacteria	10^4^
7	Enterococcus spp.	10^4^
	Skin bacteria	10^5^
8	Enterococcus spp.	10^4^
	Skin bacteria	10^3^
9	Enterococcus spp.	10^4^
	Skin bacteria	10^3^

CFU: colony forming units. Bacteriological characteristics of nine patients with false positive POC cultures from first-void urine at immediate analysis but negative POC culture from mid-stream urine.

## Discussion

### Statement of principal findings

We found that POC tests performed on MSU were significantly more accurate than when performed on FVU at immediate analysis. Delay of analysis up to four hours did not compromise the accuracy of POC tests significantly. We did not identify any patient-related factors that influenced the effect of sampling technique on agreement with the reference.

### Strengths and weaknesses of the study

The study is the first to assess how urine sampling technique from voided urine samples affects the accuracy of POC test in general practice using paired samples allowing for direct comparison.

One person performed all the analyses and we registered no missing data. Blinding was ensured as much as possible since PB was blinded to the results of the dipstick when performing microscopy and the dipstick analysis was automated for the majority of tests. Objectivity of the POC culture readings was ensured by a second reading of photographs of the POC tests by AH who was blinded to all other information.

A major limitation in this study is that the choice of reference standard could be questioned since sending urine from the MSU sample inherently introduces a bias giving MSU analysis a higher accuracy. The optimal reference standard would have been a catheter sample after obtaining the two other samples [14] or to send both the MSU and FVU sample to the microbiological laboratory. However, it is doubtful whether women would provide consent for the procedure and whether enough urine could be obtained for two boric acid containers. Since our main aim was to identify a difference between the two sampling techniques, using the MSU sample as reference proved most feasible.

There were some limitations to blinding in the study since PB was not blinded to clinical history, the sampling technique or the time-delay when interpreting the POC test. The increasing accuracy with increasing delay of analysis for POC microscopy could possibly be due to review bias since PB was not blinded to the result of the previous microscopies and the dipstick analysis when performing the subsequent microscopies [15]. This could be part of the explanation for the surprising finding that microscopy became more accurate if the analysis was performed after four hours instead of immediately.

A significant difference was found when comparing the dipstick results from the visual reading of the first 25 women vs. the Urilyzers reading of the last 92 women. However, visual reading did not significantly modify the effect of sampling technique on accuracy of urine dipstick. Thus, our main results can be interpreted without taking this limitation into account.

### Findings in relation to other studies, principally concerning differences in results

We found a difference in the diagnostic accuracy of the POC test when using MSU compared to FVU. This adds to the findings in the systematic review from Holm and Aabenhus, where they did not find consistent evidence when comparing the different sampling techniques in general practice [5]. The two studies included in the review that compared different voided techniques, were using a randomized design instead of paired samples [16,17]. When looking at sampling techniques, a paired sample results in stronger and more compareble results.

Enterococcus spp. was the the primary reason for the false positive FVU-samples. A study by Hooton et al. found the same tendency, where the MSU contained Enterococcus spp., but the catherurine did not [18]. This suggest that Enterococcus spp. is only found in the urethra and not in the bladder, and the significance of the Enterococcus as a uropathogen is questionable.

A systematic review from 2016 found that urine stored at room temperature for more than four hours resulted in overgrowth of both contaminants and significant growth [19]. In our study we found that there was no change in accuracy for POC culture and dipstick analysis when analysis was delayed up to four hours.

Accuracy measures of POC culture were quite high compared to a previous study performed in a number of practices in the Copenhagen area [20]. This difference could reflect that this study was conducted under optimal conditions and the previous study was performed in a clinical setting.

### Meaning of the study, mechanisms and implication

This study has shown that POC tests performed on MSU are more accurate than on FVU, immediately after voiding. However, in the clinical setting, analysis is often delayed, and according to our results, this does not result in a lower accuracy of the POC tests regardless of voiding technique. The main reason for the lower accuracy of POC tests performed on FVU was a small amount of false positive due to Enterococcus spp., but the clinical significance of this bacterium is not fully established. Our findings were consistent for both urine dipstick, POC microscopy and POC culture.

Based on this study we would recommend practices that use urine dipstick with or without POC microscopy, to instruct their patients to deliver a MSU sample. If the practice uses POC culture, MSU still provides the most accurate result, but if findings of Enterococcus spp. are interpreted with caution as it should, FVU can be used without compromising accuracy. Urine samples do not need to be stored in a refrigerator if analyzed within four hours.

1. Any necessary ethical approval: All procedures followed were in accordance with the Helsinki Declaration of 1975, as revised in 1983. The study was presented to the Ethical Committee for the Capital Region of Denmark and did not need ethical approval (Ref. No.: FSP 15008103). All patients gave written informed consent prior to participating in the study. The study was approved by the Danish Data Protection Agency (Ref. No.: 2015-41-4260).
2. The source of funding for the study: University of Copenhagen, 2016 funds, and The PLU fond (Praktiserende Laegers Undervisningsfond)
3. Any conflict of interest: None
4. *Registration number if clinical trial: Trial registered at:*https://clinicaltrials.gov/*Study ID: NCT02585115*
